# The role of multivitamin and mineral supplements in supporting health and well-being: a retrospective cross-sectional study in Taiwan

**DOI:** 10.1186/s41043-025-01164-y

**Published:** 2025-12-23

**Authors:** Vandana Garg, Joseph Lin, Aida Gadzhieva-Moore, Shi Mun Yee, Abhijeet Dhiman, Richa Kumari, Sheryl S. L. Tan

**Affiliations:** 1 Haleon, 23 Rochester Park, Singapore, 139234 Singapore; 2 Haleon, UK Services Limited Taiwan Branch, Taipei, 100507 Taiwan; 3 IQVIA Solutions Asia Pte Ltd, 79 Anson Road, Singapore, 079906 Singapore; 4 IQVIA Solutions Malaysia Sdn Bhd, No. 1, Jalan SS21/58, Damansara Uptown, Petaling Jaya, 47400 Malaysia; 5 Knowledge Centre, WNS Global Services, Gurugram, 122002 India

**Keywords:** Energy, Health, Immunity, Multivitamin and mineral supplements, Quality-of-life, Real-world evidence, Well-being

## Abstract

**Background:**

Multivitamin and mineral (MVM) supplements have been shown to provide various health benefits, including enhanced energy, skin health, metabolism, and immune function. This study explored the impact of MVM supplements based on users’ experience in Taiwan.

**Methods:**

This retrospective, real-world, cross-sectional study included 400 respondents, aged 35–60 years, residing in Taiwan. Data were collected via an online survey. Respondents self-reported perceived benefits across six youthful vitality benefits: energy, metabolism, sleep, mental alertness, skin health and complexion, and spirit. Additional assessments covered perceived immunity, general health, quality-of-life (QoL), well-being, user attitude and perception. Subgroup analyses was performed post hoc based on gender (male vs. female), age (< 50 vs. ≥50 years) and frequency of use (daily vs. occasional) to explore potential subgroup differences in perceived benefits.

**Results:**

A total of 93% of users agreed that MVM supplements support at least one out of six youthful vitality benefits. Users reported several perceived benefits: energy support (59.00%), improved metabolism (59.75%), improved complexion (53.25%), normal immune functioning (66.25%), confident in maintaining overall health (63.25%). Satisfaction levels were notably high, with 74.50% expressing satisfaction with product use, 72.50% trusting the product, and 79.00% showed willingness to repurchase. Subgroup analyses suggested that males, daily users, and users aged ≥ 50 years experienced more favorable outcomes across several parameters as compared to their respective counterparts.

**Conclusion:**

This study provides real-world evidence that MVM supplements are widely perceived to support youthful vitality, immunity, general health, and QoL. These user-reported outcomes complement existing clinical research by highlighting benefits experienced in everyday settings, especially among daily users and those aged 50 and above. The high satisfaction and trust levels observed further reinforce the relevance of MVM supplements as a self-care strategy. By bridging the gap between controlled trials and consumer experience, this study adds valuable context to the science and recommendation of micronutrient supplementation.

**Supplementary Information:**

The online version contains supplementary material available at 10.1186/s41043-025-01164-y.

## Introduction

Vitamins and minerals play direct or indirect roles in several metabolic pathways that support fundamental cellular functions [1–3]. Insufficient intake of essential micronutrients is a significant public health concern, affecting both adults and the elderly. Although a nutrient-rich, balanced diet can provide essential vitamins and minerals, factors like food availability and personal choices limit their intake [3, 4]. Furthermore, increased consumption of energy-dense, processed, fried, and animal-based products, along with sugar-sweetened drinks and salty snacks, has reduced micronutrient intake across several Asian countries [5, 6]. In Taiwan, the healthy Taiwanese eating approach which promotes higher intake of plant-based and aquatic food is generally recommended. This is associated with lower mortality rate, improved well-being, and healthy longevity [7, 8]. However, only 20–30% of the Taiwanese population adhere to these recommended dietary guidelines [9]. Consequently, a significant portion of the population fails to meet their optimal vitamin and mineral requirements through diet alone, leading to widespread micronutrient inadequacy [3]. Implementing nutrition education and appropriate strategies is necessary to address this inadequacy [10–13].

According to the National Academic Council of Taiwan reports, a substantial increase in the proportion of the elderly population (>65 years) has turned Taiwan into an aged society, and it is predicted to become a super-aged society by 2025 [14]. Data from the Nutrition and Health Survey in Taiwan, 2014–2017 has indicated an association between nutrient inadequacies and conditions like frailty and cognitive impairment among the elderly [15]. Maintaining optimal micronutrient status throughout life is crucial for supporting healthy homeostasis among ageing population [16]. Inadequate levels of micronutrients can impair various physiological processes, even in healthy adults [2]. Multivitamin and mineral (MVM) supplements are commonly used to address this micronutrient imbalance. Beyond preventing micronutrient inadequacy, MVM supplements have the potential to support the physical health of older adults [17]. Scientific evidence have emphasized that supplements are commonly used to address dietary imbalances, prevent joint degeneration and osteoporosis, reduce mental and physical fatigue, improve concentration, and boost energy and immunity [2, 17, 18]. Micronutrients are crucial for transformation of energy sources or macronutrients into cellular energy [3, 19]. Furthermore, systematic reviews and meta-analyses have linked adequate micronutrient levels with better sleep patterns [20], as well as positive effects on perceived stress, mood, and mild psychiatric symptoms in healthy individuals [21, 22]. Consequently, MVM supplements, combined with a balanced diet, can play a significant role in enhancing quality-of-life (QoL) [23]. In the past two decades, the consumption of health supplements has surged in several Asian countries. Vitamins and mineral supplements are among the most commonly consumed dietary supplements in Taiwan [17, 24], with MVM supplements intake almost doubled (23.6% to 42.3%) from 1993-1996 to 2005–2008 [25]. A cross-sectional study conducted in Malaysia reported a 43% prevalence of vitamin-mineral supplement use [26]. Additionally, 63% of subjects in Indonesia and 76.10% in Thailand use food or dietary supplements [27, 28]. The motivation for supplement use varies, including enhancing mental resilience, supporting lifestyle changes, maintaining energy levels and good health, recovering from illness, and managing stress [2, 26].

As supplement usage continues to increase, it is important to understand existing users’ perspectives towards supplement use and outcomes. This retrospective cross-sectional study investigated the real-world impact of MVM supplement Centrum (hereafter referred to as MVM) use among users in Taiwan, focusing on user-reported outcomes. The investigation prioritized six key benefits such as energy, metabolism, sleep, mental alertness, skin health and complexion, and spirit, all of which collectively contribute to youthful vitality. Additionally, the study evaluated perceived benefits regarding immunity, general health, well-being, and QoL associated with MVM supplements use. To provide deeper insights, outcomes were stratified by age, gender, and frequency of MVM supplements use. By exploring user satisfaction and trust, this study aimed to provide valuable insights on the role of MVM supplements in enhancing youthful vitality and various aspects of health in real-world settings. In addition to clinical studies, real-world studies provide further insights on how interventions such as MVM supplements perform in diverse everyday health settings, beyond the controlled environments of clinical trials. These insights are critical for shaping public health initiatives and empowering individuals to make informed decisions regarding MVM supplement use.

## Methodology

### Study design

This retrospective, real-world, cross-sectional study in Taiwan, used real-life data to evaluate perceived benefits and satisfaction of existing MVM supplements users. Respondents completed a self-administered, structured online questionnaire. Informed consent was obtained from all respondents prior to the survey. This study did not require ethics committee approval because of its cross-sectional study design, involved no healthcare providers or any intervention, accessed no medical records [2] .

### Study population

Existing users of MVM supplements, who met the inclusion criteria, were invited to complete the survey. The key inclusion criteria to participate in the survey were: (1) male or female residents of Taiwan aged 35 to 60 years old, (2) respondents must be existing users of Centrum Advance or Centrum Silver (Men or Women variants), (3) respondents must have used Centrum MVM supplements at least 3 times a week over the past 3 months, and (4) self-report as healthy. Respondents with serious chronic illnesses such as cancer, Crohn’s disease, multiple sclerosis, epilepsy, or HIV/AIDS within the past 12 months, were excluded. Respondents who used other supplement brands 3 times per week or more in combination with the regular use of Centrum MVM supplements were excluded.

### Data collection and assessment

A unique ID was assigned to each survey respondent to ensure anonymity throughout the study. Respondents were given a predetermined set of questions that were designed to be consumer-friendly, self-explanatory, and free of technical language. To ensure the survey was accessible and easily understood by a general audience, the use of plain language was emphasized, deliberately excluding technical or scientific content that the study team identified as potentially difficult for the average consumer to understand. Questions were developed using everyday vocabulary and clear sentences. The survey was designed to be self-explanatory, with intuitive structure, clear instructions, and logical flow. Multiple internal reviews were conducted to assess clarity and ensure that the language was appropriate for a broader consumer audience in Taiwan. The survey questions and informed consent were provided to respondents in Traditional Chinese (Taiwan) language, verified by a native speaker to ensure accuracy within the study context.

In the survey, respondents provided information on gender, age, frequency of use, perceived contributors to youthful vitality and reasons for MVM supplements use. The construct of youthful vitality used in the study is a novel measure developed to capture multidimensional perceptions of well-established health domains viz. energy (vitality), metabolism, sleep, mental alertness, skin health, and spirit among supplement users. While it is not directly derived from previously validated scales, its conceptual foundation aligns with vitality-related domains assessed by established instruments such as the SF-36 vitality scale and WHO-5 well-being index, or with consumer-friendly statements directly mapping onto those domains. Youthful vitality was operationalized through specific survey items informed by prior consumer insights and pilot-tested during the Core MVM Relaunch Concept Test in Taiwan. This process identified six vitality dimensions i.e. energy, spirit, complexion, metabolism, sleep, and mental alertness as relevant to the target population. These findings support the internal consistency and discriminant validity of this construct. Respondents rated their level of agreement with the custom-designed statements assessing perceived benefits following MVM supplements usage. Energy and performance, metabolism, sleep, mental alertness, skin health and complexion, QoL, immunity and general health were rated on a 5-point scale [25]. Energy level and perceived benefits regarding well-being were rated on a 6-point scale.

The 5-point scale used for rating energy and performance, metabolism, sleep, mental alertness, skin health and complexion, immunity and general health, and QoL was as follows; 1: strongly disagree; 2: disagree; 3: neither agree nor disagree; 4: agree; 5: strongly agree. Respondents completed health-related assessments using scientifically validated self-report tools to examine the perceived benefits of MVM supplements on different health parameters. This includes the Immune Status Questionnaire (ISQ) [29], the vitality scales from the RAND 36-Item Health Survey 1.0 [30], and the World Health Organization-Five Well-Being Index (WHO-5) [31].

The ISQ is a self-assessment tool designed to measure perceived immunity status based on the frequency of immune-related complaints experienced in the past 12 months. The ISQ comprises of seven items including ‘sudden high fever’, ‘diarrhea’, ‘headache’, ‘skin problems (e.g., acne and eczema)’, ‘muscle and joint pain’, ‘common cold’ and ‘coughing’. Respondents report the frequency of each health complaint on a 5-point scale (0: never; 1: sometimes; 2: regularly; 3: often; 4: almost always). The derived ISQ score ranges from 0 (very poor) to 10 (excellent). ISQ score of ≥ 6 is indicative of normal immune functioning, while a score of < 6 suggests reduced immune functioning based on the development and validation of ISQ [29].

The 36-Item Short-Form Health Survey (SF-36) vitality scale was employed to measure the perceived energy levels among MVM supplements users. This scale assesses how often a person feels energetic, full of pep, worn out or tired over the past 4 weeks, on a 6-point scale (1: all of the time; 2: most of the time; 3: a good bit of the time; 4: some of the time; 5: a little of the time; 6: none of the time). The RAND method was used to score the items. These responses are transformed to derive an overall vitality score ranging from 0 to 100, where higher scores reflect greater energy levels [30].

The WHO-5 is a self-reported questionnaire to evaluate subjective mental well-being. It consists of five statements relating to well-being, which respondents rate based on their experiences over the past two weeks. Each statement is scored on a 6-point scale (0: at no time; 1: some of the time; 2: less than half of the time; 3: more than half of the time; 4: most of the time; 5: all the time). The total score ranges from 0 to 25, where 0 represents the worst possible well-being, and 25 represents the best possible well-being [25, 32]. Scores of ≥ 13 indicate normal well-being, while a score of < 13 indicates poor well-being [31].

Respondents rated their perceived trust, satisfaction, likelihood to repurchase, and willingness to recommend MVM supplements to others, including family and friends, using a 5-point scale. For parameters scored on a 6-point scale, the outcome was calculated as the aggregated percentage of the top three ratings (‘All the time’, ‘Most of the time’, and ‘More than half of the time’/ ‘A good bit of the time’). For parameters scored on a 5-point scale, the outcome is reflected as the aggregated percentage of the top two ratings (‘Agree’ and ‘Strongly agree’). Subgroup analysis was conducted based on gender (male vs. female users), age (≥ 50 years vs. <50 years), and usage frequency (daily vs. occasional users). Daily users consumed MVM supplements every day, whereas occasional users consumed them 3–5 days per week.

### Sample size, data, and statistical analysis

The sample size was calculated using Cochran’s formula [33]. Assuming a 5% error margin, a standard deviation (SD) of 0.5, and a 95% confidence level (CI), a sample size of approximately 400 respondents was deemed sufficient for this digital survey. Only completed questionnaires were included in the analysis. All continuous variables were expressed as mean ± SD, while categorical variables were presented as percentage (%). Comparisons between groups were done post hoc using the paired/overlap z-test for categorical variables and the paired/overlap t-test for continuous variables. Differences between groups were considered significant at the 5% level (*p* < 0.05). As this was an exploratory study and the analyses were not intended to test confirmatory hypotheses, no adjustments for multiple testing were applied. The primary aim was to explore potential subgroup differences that could guide future research. All data management and analysis were performed using WinCross version 21.0 and IBM SPSS Statistics software, version 23.0.

## Results

### Demographics and other characteristics

A total of 400 respondents completed the questionnaire. The gender distribution was nearly equal, including 50.25% male and 49.75% female users, with a mean age of 46.72 ± 6.80 years. The majority of respondents (64.25%) were below 50 years old. A total of 59.25% respondents were daily users of MVM supplements while 40.75% were occasional users (Supplementary Table S1).

**Fig. 1:**
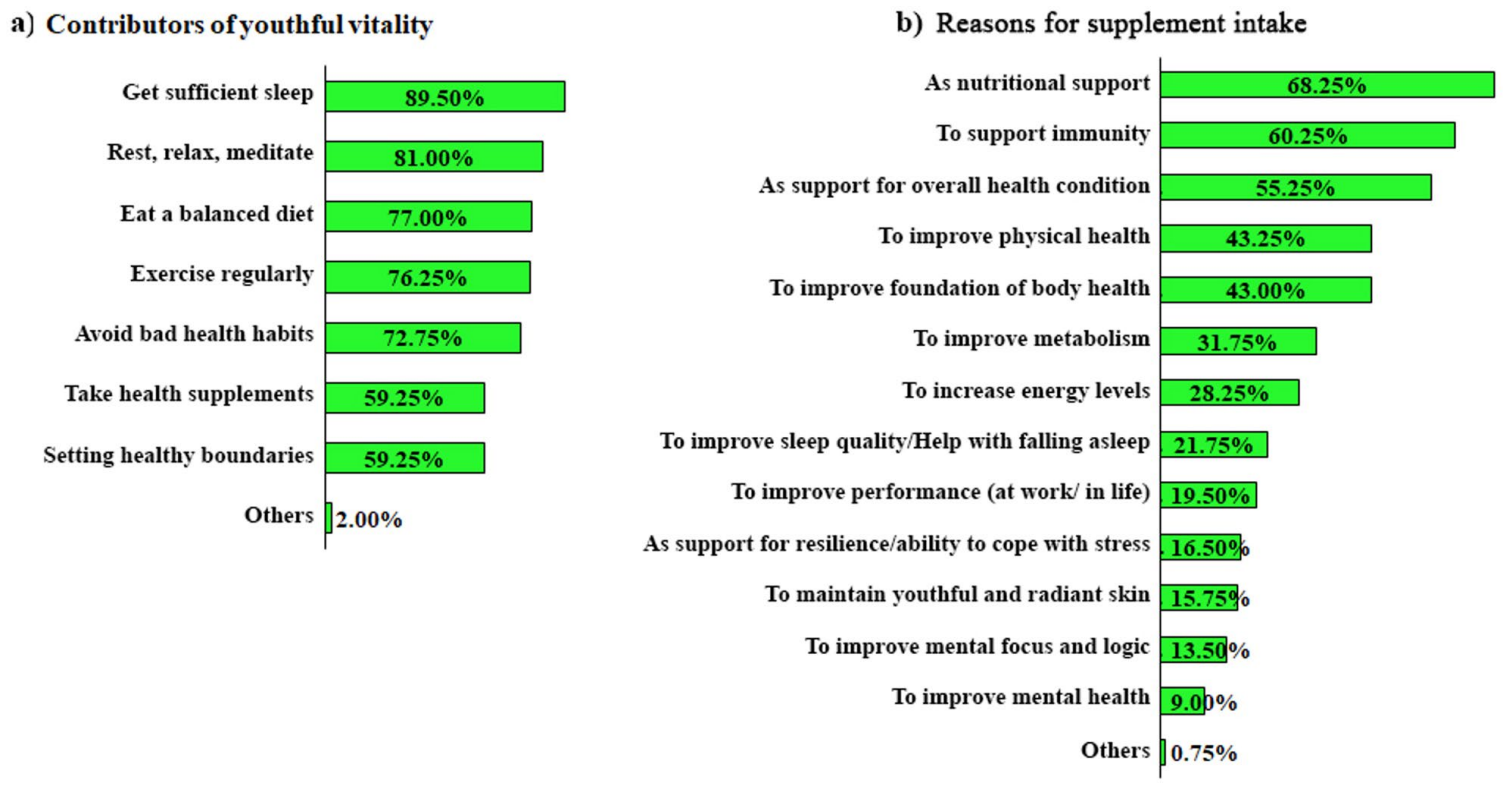
(**a**) Perceived contributors of youthful vitality, (**b**) Reasons for supplement intake

Among the 400 respondents, around 59.25% recognize health supplements as a key contributor to youthful vitality (Fig. 1a). MVM supplements were primarily taken as nutritional support (68.25%), immunity support (60.25%), and to support overall health conditions (55.25%) (Fig. 1b). Moreover, 93.00% of respondents reported that MVM supplements support their youthful vitality, with perceived benefits in at least one of the six aspects: energy, metabolism, complexion, sleep, mental alertness, and spirit (Supplementary Table S2).

### Perceived benefits of MVM supplements in supporting youthful vitality

#### Energy-related benefits

Users’ experience pertaining to the benefits of MVM supplements on energy levels and performance are summarized in Table 1. Among the 400 users, most of them perceived that MVM supplements give them energy to perform better at day-to-day life (59.00%), while 50.50% reported that they have more energy for sports and training. Around 49.00% of users reported that MVM supplements give them good energy all day long, with a significantly higher percentage of male users compared to female (54.23% vs. 43.72%; *p* = 0.034) perceiving this benefit (Supplementary Table S3).

**Fig. 2:**
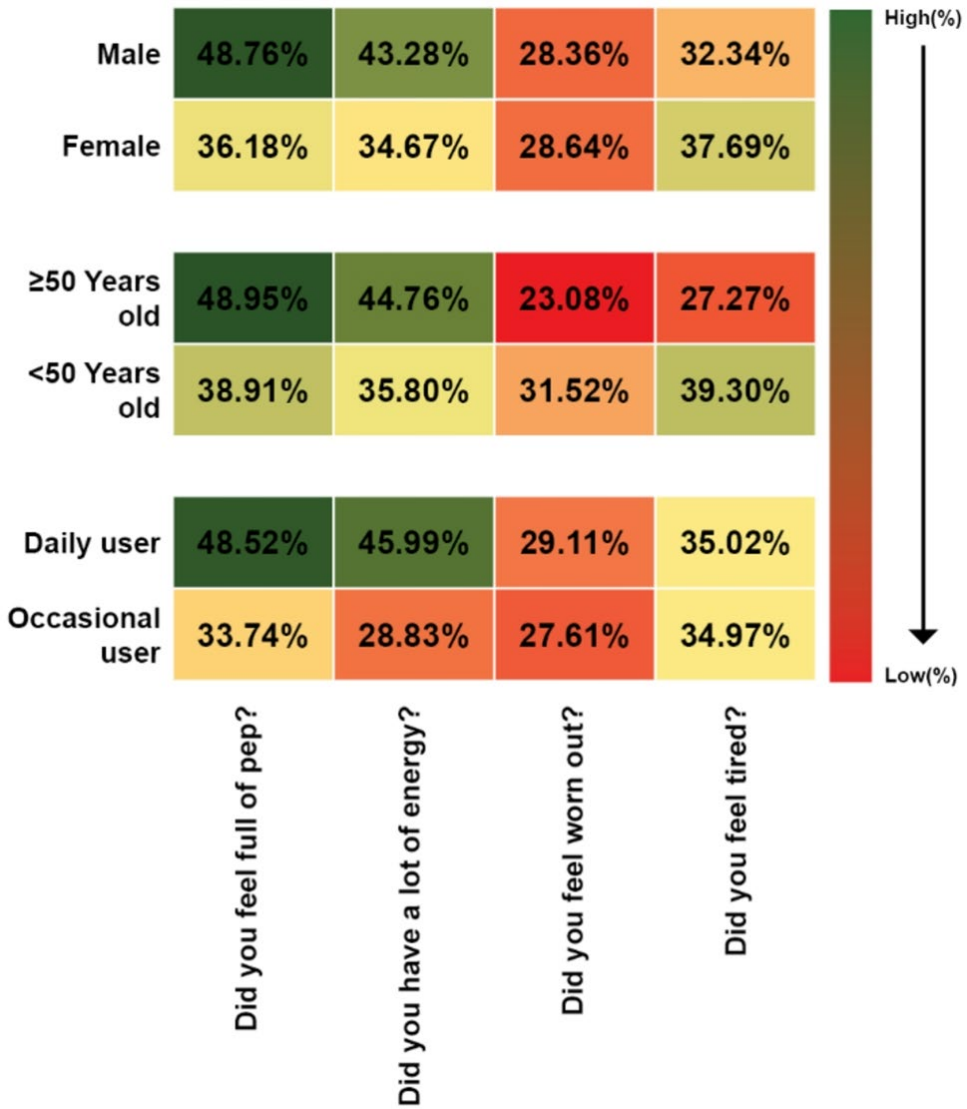
Perceived frequency of experience pertaining to energy-related statements from the SF-36 vitality scales. The perceived frequency of experience (%) is derived from the aggregated percentage score of those who chose ‘All of the time’, ‘Most of the time’ and ‘A good bit of the time’ for each statement. Perception in different subgroups is represented as three-color gradient heat map. Green color indicates higher aggregated % while red color represents lower aggregated %

Item analysis regarding statements on energy level from SF-36 vitality scales showed that 42.50% of users reported feeling full of pep, 39.00% indicated having a lot of energy. Conversely, 35.00% reported feeling tired, and 28.50% described feeling worn out (Supplementary Table S4). The detailed responses stratified by gender, age, and frequency of MVM supplements use, are presented as heatmap in Fig. 2. A significantly higher proportion of male as compared to female users (48.76% vs. 36.18%; *p* = 0.010), and daily compared to occasional users (48.52% vs. 33.74%; *p* = 0.003) reported “feeling full of pep”. Although not statistically significant but a higher percentage of individuals aged ≥ 50 years (48.95%) reported “feeling full of pep” compared to those < 50 years (38.91%). Furthermore, a higher percentage of males (43.28%) and individuals aged ≥ 50 years (44.76%) reported “having a lot of energy” compared to female users (34.67%) and those < 50 years (35.80%), respectively. Additionally, a significantly higher percentage of daily users also reported “having a lot of energy” compared to occasional users (45.99% vs. 28.83%; *p* = 0.0004).

The SF-36 vitality scale was used to assess overall perceived energy levels. The mean vitality score of the total sample was 51.48 ± 17.84. This score was significantly higher in daily users compared to occasional users (52.87 ± 19.44 vs. 49.45 ± 15.05; *p* = 0.048), and in individuals aged ≥ 50 years compared to those < 50 years (54.27 ± 18.69 vs. 49.92 ± 17.19; *p* = 0.023) (Supplementary Table S5).

#### Metabolism-related benefits

Among the 400 users, 59.75% reported that MVM supplements improved their metabolism (Table 1). This perceived benefit was significantly more common among male as compared to female users (65.17% vs. 54.27%; *p* = 0.025) (Supplementary Table S6).

#### Skin health and complexion-related benefits

A total of 53.25% users perceived that MVM supplements improve their overall complexion (Table 1). This benefit was perceived more commonly among individuals aged ≥ 50 years compared to those < 50 years (60.14% vs. 49.42%; *p* = 0.037). Additionally, 38% of users reported that their skin looks better while taking MVM supplements, with a significantly higher percentage of users aged ≥ 50 years compared to < 50 years (45.45% vs. 33.85%; *p* = 0.023) (Supplementary Table S7) perceiving this benefit.

#### Sleep-related benefits

Over half of the users (57.50%) perceived that they wake up feeling more energetic to start the day. Additionally, 41.50% agreed that MVM supplements helped them fall asleep, so they wake up with a good mood the next day (Table 1).

#### Mental alertness and spirit-related benefits

A total of 45.75% users perceived improved mental performance, so that they can pay more attention to their day-to-day tasks. Furthermore, 41.25% of users agreed that they felt more mentally focused while taking MVM supplements (Table 1). Notably, 53.25% of users reported that they can maintain a good spirit while using MVM supplements (Supplementary Table S2).

**Table 1:** Perceived benefits of MVM supplements pertaining to different youthful vitality parameters

Youthful vitality statements		Users’ agreement (%), *n* = 400					
		Top 2 ratings (Strongly agree + Agree)	Strongly agree	Agree	Neither agree nor disagree	Disagree	Strongly disagree
Energy and performance benefit							
	MVM supplements give me good energy all day long.	49.00%	5.25%	43.75%	44.50%	6.50%	0.00
	MVM supplements give me energy to perform better at my day-to-day life.	59.00%	6.75%	52.25%	37.50%	3.50%	0.00
	With MVM supplements, I feel more energetic to engage in my personal hobbies.	47.75%	5.50%	42.25%	47.25%	4.50%	0.50%
	With MVM supplements, I find I have more energy for sports/ training.	50.50%	6.25%	44.25%	44.00%	5.25%	0.25%
Metabolism							
	With MVM supplements, my metabolism has improved.	59.75%	7.25%	52.50%	35.50%	4.25%	0.50%
Skin health and complexion							
	MVM supplements improves my overall complexion	53.25%	5.25%	48.00%	43.00%	3.25%	0.50%
	My skin looks better while taking MVM supplements	38.00%	5.00%	33.00%	55.25%	6.25%	0.50%
	My skin looks healthier while taking MVM supplements	36.25%	4.00%	32.25%	56.25%	6.50%	1.00%
	MVM supplements improve my overall skin health.	36.50%	3.50%	33.00%	55.75%	7.50%	0.25%
Sleep							
	I started to fall asleep easier with MVM supplements use.	32.00%	3.00%	29.00%	55.50%	10.00%	2.50%
	With MVM supplements, I wake up feeling more energetic to start the day.	57.50%	5.00%	52.50%	38.25%	3.50%	0.75%
	MVM supplements helps me fall asleep, so I wake up with a good mood next day.	41.50%	4.00%	37.50%	49.25%	7.50%	1.75%
Mental alertness							
	I am more mentally focused while taking MVM supplements.	41.25%	4.75%	36.50%	52.00%	6.25%	0.50%
	With MVM supplements, I can have better memory.	35.50%	4.25%	31.25%	56.75%	7.25%	0.50%
	With MVM supplements, my mental performance has improved, so that I can pay more attention to my day-to-day tasks.	45.75%	4.75%	41.00%	48.25%	5.75%	0.25%
	MVM supplements helps me stay mentally alert, so I can learn more new things.	38.25%	3.75%	34.50%	53.75%	7.50%	0.50%
	MVM supplements helps me stay focused, so I can be more organized.	39.50%	4.50%	35.00%	52.50%	7.25%	0.75%

### Perceived benefits of MVM supplements in supporting general health, well-being and QoL

#### Immunity- and general health-related benefits

The perceived immunity and general health benefits of MVM supplements were analyzed. It was perceived that 68.75% of users stay in healthy condition and 62.75% users agreed that MVM supplements help them lay a good foundation for their body health. Additionally, 67.00% of respondents felt that their body defense has improved while taking MVM supplements (Table 2).

**Table 2:** Perceived benefits of MVM supplements pertaining to immunity and general health

Statements on perceived immunity and general health	Users’ agreement (%), *n* = 400					
	Top 2 box (Strongly agree + Agree)	Strongly agree	Agree	Neither agree nor disagree	Disagree	Strongly disagree
MVM supplements helps me lay a good foundation for my body health.	62.75%	7.25%	55.50%	34.00%	2.75%	0.50%
MVM supplements makes me feel the best from inside out.	56.75%	7.00%	49.75%	40.50%	2.75%	0.00%
I stay in healthy condition while taking MVM supplements.	68.75%	7.25%	61.50%	28.50%	2.75%	0.00%
My body defense improved while taking MVM supplements.	67.00%	7.75%	59.25%	29.75%	3.25%	0.00%
With MVM supplements, I can get proper nutrition to get back to healthy condition after sickness.	45.00%	5.25%	39.75%	47.25%	6.50%	1.25%
MVM supplements help support my heart health.	35.25%	5.50%	29.75%	55.25%	8.50%	1.00%

The immune functioning status of MVM supplements users is presented in Supplementary Table S8. A mean ISQ score of 6.44 ± 2.22 was observed, representing normal immune functioning (≥ 6). Notably, males had significantly higher mean ISQ scores compared to females (6.78 ± 2.31 vs. 6.09 ± 2.06; *p* = 0.002). Similarly, MVM supplements users aged ≥ 50 years reported significantly higher scores than those aged < 50 years (6.80 ± 2.21 vs. 6.24 ± 2.20; *p* = 0.016). Furthermore, daily users also reported higher score than occasional users.

#### Well-being-related benefits

Item analysis from WHO-5 well-being tool is presented in Table 3. A total of 64.50% of users felt calm and relaxed, while 60.00% reported feeling cheerful and in good spirits. The mean WHO-5 well-being score was recorded as 13.23 ± 5.20, indicating normal mental well-being. Notably, mean well-being score for daily users (13.89 ± 5.45 vs. 12.26 ± 4.65; *p* = 0.001) and those ≥ 50 years old (13.94 ± 5.21 vs. 12.84 ± 5.16; *p* = 0.043) was significantly higher compared to their respective counterparts, indicating a more favorable well-being outcome (Supplementary Table S8).

**Table 3:** Detailed answers of the WHO-5 tool

Statements on WHO-5 well-being	Users’ agreement (%), *n* = 400						
	Top 3 Box (All the time + Most of the time + More than half of the time)	All the time	Most of the time	More than half of the time	Less than half of the time	Some of the time	At no time
I have felt cheerful and in good spirits	60.00%	1.75%	27.25%	31.00%	22.75%	15.75%	1.50%
I have felt calm and relaxed	64.50%	3.50%	24.25%	36.75%	19.25%	15.25%	1.00%
I have felt active and vigorous	56.75%	2.75%	21.50%	32.50%	21.50%	18.25%	3.50%
I woke up feeling fresh and rested	54.25%	2.00%	21.75%	30.50%	22.75%	18.50%	4.50%
My daily life has been filled with things that interest me	57.75%	3.00%	21.50%	33.25%	20.00%	20.75%	1.50%

#### QoL-related benefits

The influence of MVM supplements on QoL is summarized in Table 4. Confidence in maintaining overall health with one pill a day was reported by 63.25% of users. Additionally, 49.50% reported that their QoL has improved due to MVM supplements, with a significantly higher percentage of male users reported improvements compared to female users (56.22% vs. 42.71%; *p* = 0.006) (Supplementary Table S9).

**Table 4:** Perceived benefits of MVM supplements pertaining to quality-of-life

Statements on Quality-of- life	Users’ agreement (%), *n* = 400					
	Top 2 box (Strongly agree + Agree)	Strongly agree	Agree	Neither agree nor disagree	Disagree	Strongly disagree
My quality-of-life improved with MVM supplements.	49.50%	4.75%	44.75%	45.00%	4.75%	0.75%
With MVM supplements, I am taking good care of my body and mind.	60.50%	6.75%	53.75%	35.50%	3.25%	0.75%
MVM supplements supports me to keep a healthy and active lifestyle and enjoy activities I love.	53.75%	5.25%	48.50%	43.00%	3.00%	0.25%
With just 1 pill a day, MVM supplements gives me confidence to maintain my overall health.	63.25%	6.00%	57.25%	32.50%	3.75%	0.50%

### Attitude and perception of users’ towards MVM supplements

**Fig. 3:**
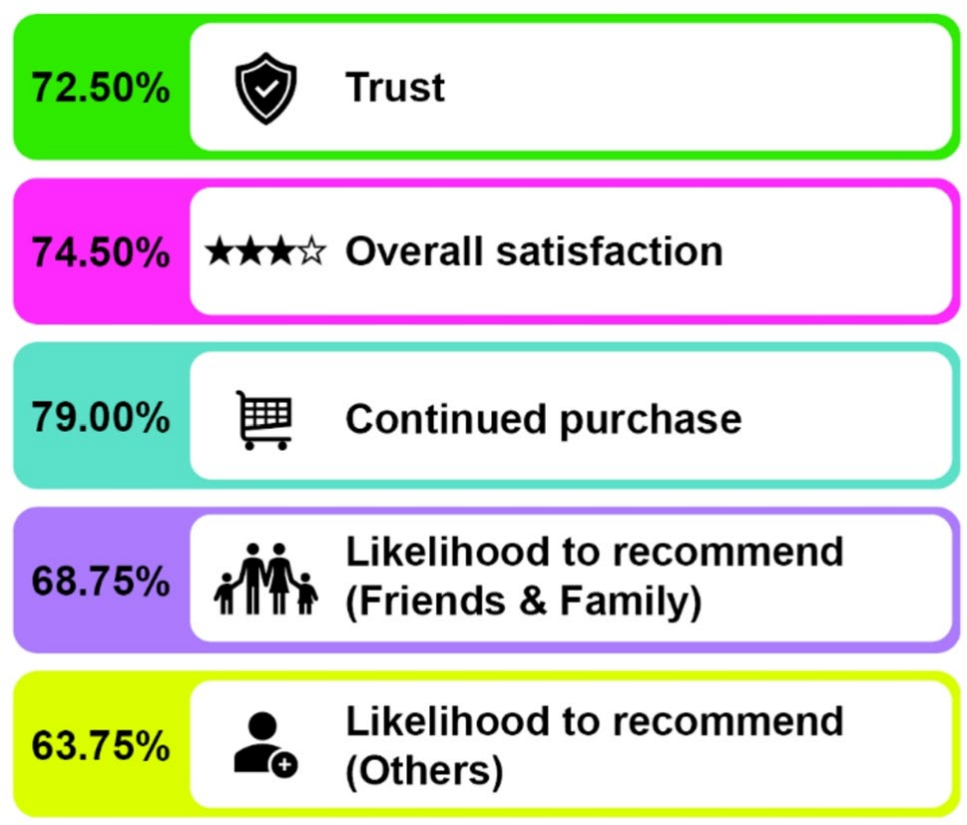
User attitudes and perception towards MVM supplements

The analysis of users’ attitude and perception towards MVM reported favorable outcome across multiple parameters (Fig. 3 and Supplementary Table S10). Majority of users (72.50%) reported trust in the product, indicating a strong perception of reliability. Overall satisfaction was even higher (74.50%), reflecting positive experience with the product. Importantly, 79.00% of users indicated that they would buy the product again, underscoring a high level of continued purchase interest. When it comes to recommending the product, 68.75% of users stated that they would recommend it to friends and family, while 63.75% expressed a likelihood of recommending it to others.

## Discussion

This real-world evidence study explored the experience and usage pattern of MVM supplements users among healthy population in Taiwan, offering valuable user-reported insights that complement findings from clinical trials. MVM supplement is defined as any product that contains three or more vitamins and minerals, with each nutrient at levels below the tolerable upper limit set by the Food and Nutrition Board and it excludes herbs, hormones, or drugs [34]. While randomized trials primarily assess the effects of MVM supplements on clinical outcomes, this study explored the perceived benefits in daily life. Several outcomes indicated potential differences based on gender, age and frequency of use which can be further validated through future research. The findings of the study indicated that over 90% users believe that MVM supplements support their youthful vitality, with perceived benefits in at least one out of the six parameters: energy, metabolism, sleep, mental alertness, skin health and complexion, and spirit, underscoring the broad utility of MVM supplements.

More than one-third of MVM supplements users in this study reported feeling a lot of energy and full of pep. Additionally, over half indicated that these supplements provided energy support for sports, training and to perform better at their day-to-day life, as well as improved metabolism. These findings emphasize the role of MVM supplements in supporting energy and metabolism. Micronutrient deficiencies can disrupt the biochemical processes, limiting energy production and can potentially lead to metabolic and health issues [3, 19]. MVM supplements help improve blood concentrations of various vitamins and support metabolic processes by increasing cellular oxygen consumption and basal metabolic rates [35]. The B vitamins, in particular, are critical for mitochondrial function and energy production, playing a key role in mitochondrial respiration, the tricarboxylic acid cycle, the electron transport chain, ATP synthesis, and one-carbon metabolism [19, 36]. Along with B-vitamins, vitamin C, iron and magnesium play roles in different steps of energy-yielding metabolism [3]. Kennedy et al. reported that acute micronutrient supplementation can modulate energy metabolism, with sustained benefits observed during chronic use [1]. Findings of this study suggest that reported benefits related to energy varied across gender, age and frequency of use. A higher percentage of male users, individuals aged 50 years and older, and daily users reported feeling energetic and full of pep, as compared to their respective counterparts. A greater percentage of male users also perceived improvements in metabolism, aligning with a previous study that MVM supplements increases carbohydrate oxidation and energy expenditure in males [37]. Notably, even a single daily dose of MVM supplement can influence energy expenditure and reduce subjective tiredness, highlighting their immediate effects. The observed difference between male and female users suggest that gender may significantly influence MVM supplements’ impact on energy metabolism [37]. As individuals age, their intake of both macronutrients and micronutrients tends to decrease, often resulting in insufficient nutritional status. Older adults who may be at a higher risk of micronutrient deficiencies due to reduced dietary intake, reported benefits from MVM supplements. The likely reason is the improvement in micronutrient levels from MVM supplements [35], which may positively enhance their overall health and well-being.

Vitamins and their derivatives also play a crucial role in maintaining skin health, contributing to keratinocyte normalization, antioxidant activity, collagen formation, melanogenesis inhibition, and anti-inflammatory effects [38, 39]. In this study, 53.25% users reported improvement in overall complexion, with a significantly higher percentage of users aged 50 years and above (60.14%) perceiving this benefit as compared to those below 50 years (49.42%). Previous research has also demonstrated significant improvement in skin appearance with vitamins and minerals supplements compared to placebo [40]. With MVM supplements, 41.50% users reported that it helps them fall asleep, so they wake up with a good mood next day. Furthermore, 57.50% users reported waking up feeling more energetic to start the day. Micronutrients like magnesium, zinc, and vitamin D have been shown to improve sleep quality by calming the nervous system and regulating neurotransmitters and hormones involved in the sleep-wake cycle [41]. A systematic review has shown that micronutrients such as iron and magnesium can positively influence neurotransmitter’s function and circadian rhythms [20].

Users also experienced improvements in mental performance (45.75%), memory (35.50%), and QoL (49.50%) while taking MVM supplements. Significantly more male users perceived QoL improvement than females, while daily users and those aged 50 years or above had significantly higher WHO-5 scores than their respective counterparts, indicating better perceived well-being. These findings are consistent with earlier research demonstrating significant mood benefits and reduced mental fatigue among MVM supplements users [37, 42, 43]. B-vitamins, vitamin C, vitamin K, iron, magnesium, and zinc improve mental fatigue and supports cognitive or psychological functions [3]. Daily use of multivitamin supplements in older adults significantly improves memory and helps maintain cognitive health [44–46]. Moreover, MVM supplements have also been associated with consistent reductions in anxiety, perceived stress, tiredness, and improved concentration [47]. These results highlight the potential of MVM supplements as a valuable approach in improving overall health and QoL, particularly in older adults.

The mean ISQ score of MVM supplements users indicated normal immune functioning, suggesting that use of MVM supplements supports the body’s ability to fight infections and maintain overall immune health. Higher ISQ scores indicated better perceived immune functioning, particularly in males and among users aged 50 years and above. Additionally, 67.00% users reported that their body defense improved while taking MVM supplements. A previous study by Barringer et al., reported fewer infection-related illnesses among MVM users compared to placebo [48]. Key vitamins and minerals, especially vitamin C, zinc, and selenium, are known to play a vital role in modulating immune response and reducing inflammation [49]. Regular multivitamin supplements in older adults have been shown to significantly decrease the duration and severity of illnesses [50]. Although no established guideline exists for MVM supplements use, the European Union has approved health claims for vitamins and minerals, acknowledging benefits for energy, metabolism, hair, skin, psychological health, and immune function [51]. Attitude and perception towards MVM supplements collectively emphasize the strong trust, satisfaction, and intent to re-purchase among existing users in this study while identifying opportunities to enhance broader recommendation appeal.

This study’s strengths include its large sample size and its reflection of real-world usage of MVM supplements. The real-world data presented here provide valuable insights into how a product functions in everyday life in a self-care setting, reflecting actual usage free from the limitations of controlled trials. Real-world evidence bridges the gap between controlled trial outcomes and actual consumer experiences, offering valuable insights into product utility. In this study, the existing users chose to purchase and use MVM supplements on their own, without any third-party intervention or prescription. Therefore, the self-reported results can be considered neutral and unbiased.

The study has certain limitations typical of real-world investigations that rely on retrospective data collected through an online questionnaire. In this setting, data monitoring is not feasible, and responses may be influenced by recall bias [52, 53]. To mitigate this, users included in the study were required to have used MVM supplements within the past three months, a timeframe deemed sufficient to minimize recall bias and ensure reliable data. Existing users were selected from a panel of individuals willing to participate in online surveys, which may limit the generalization of the findings to the broader population. Absence of a control group (non-users) limits the ability to attribute observed differences exclusively to MVM supplements. In the absence of a control group, placebo effect, expectation bias, and social desirability bias cannot be ruled out which could have otherwise provided a more robust comparison to evaluate the effectiveness of MVM supplements. Although stringent inclusion criteria (at least 3 times a week over the past 3 months) and validated instruments (ISQ, SF-36, WHO-5) were employed to enhance data robustness, no random sampling or external data sources were available to fully mitigate these biases. Confounders such as socioeconomic status, lifestyle-related factors might have affected the respondents’ perception and introduced bias, thus limiting the internal validity and possibility that findings of the study attributable to external influences. Since, the primary objective of the study was to assess the self-reported effectiveness of MVM supplements in a real-world setting, controlling for these confounders was beyond the scope of the present analysis. Expanding the study to more diverse populations and extending follow-up periods may provide deeper insights into the long-term use of MVM supplements and their broader implications for health and well-being. Given the exploratory nature of this study, subgroup comparisons were conducted post hoc to explore potential differences in perceived benefits rather than to confirm pre-defined hypotheses. Hence, adjustments for multiple testing were not applied to avoid obscuring potentially meaningful patterns that could inform future confirmatory research. While some subgroup differences (e.g. WHO-5 well-being scores) are statistically borderline, these findings are exploratory in nature and may not represent substantial clinical effects. These marginal differences should be interpreted with caution and considered as preliminary insights within the context of a self-reported, cross-sectional survey. Confirmatory studies are warranted with pre-specified hypotheses and appropriate statistical controls to assess their practical health impact. Lastly, the composite construct youthful vitality has not itself undergone formal psychometric validation. Future research should formally evaluate the reliability and validity of this multi domain measure either by factor analysis across diverse populations or by correlating it with objective biomarkers of aging and vitality.

## Conclusion

The study validates the perceived benefits of MVM supplements among existing users. Over 90% of users experienced at least one youthful vitality benefit related to energy, metabolism, sleep, mental alertness, skin health and complexion, and spirit. They also valued MVM supplements for supporting immunity, general health, QoL, and well-being. Daily users and users aged 50 and above reported comparatively better outcomes, emphasizing the value of routine MVM supplements use for sustained health benefits. Overall, users’ perception was positive, with over 70% expressing satisfaction, trust, and a willingness to repurchase. These findings offer real-world evidence that MVM supplements effectively bridge nutritional gaps, support immunity, and improve QoL. As a key element of self-care, MVM supplements support proactive health management in today’s fast-paced and demanding world.

## Supplementary Information

Below is the link to the electronic supplementary material.

Supplementary Material 1

## Data Availability

The data underlying this article will be shared on reasonable request to the corresponding author.

## Abbreviations

CI: Confidence level
ISQ: Immune Status Questionnaire
MVM: Multivitamin and mineral
QoL: Quality-of-life
SD: Standard deviation
SF-36: 36-Item Short-Form Health Survey
WHO-5: World Health Organization-Five

## References

[1] Kennedy DO, Stevenson EJ, Jackson PA, Dunn S, Wishart K, Bieri G, et al. Multivitamins and minerals modulate whole-body energy metabolism and cerebral blood-flow during cognitive task performance: a double-blind, randomised, placebo-controlled trial. Nutr Metab (Lond) Engl. 2016;13:11. 10.1186/s12986-016-0071-4 PMC4750202 26870152

[2] Moroni B, Óvári V, Nicastro C, Salvo Rd, Ehret A. A real-world evidence study evaluating consumer experience of Supradyn recharge or Supradyn magnesium and potassium during demanding periods. Drugs Context Engl; 2023;12, 2023-1-6. 10.7573/dic.2023-1-6 PMC10259500 37313041

[3] Tardy A-L, Pouteau E, Marquez D, Yilmaz C, Scholey A. Vitamins and minerals for Energy, fatigue and cognition: A narrative review of the biochemical and clinical evidence. Nutrients Switz; 2020;12:228. 10.3390/nu12010228 PMC7019700 31963141

[4] Phenias N, Tariku GB, Kate L, Hilda V, Stefaan DH. Exploratory dietary patterns, the global diet quality score, and their associated socio-demographic factors among young adults in rwanda: a cross-sectional study using a food list-validated, semi-quantitative food frequency questionnaire. Nutr Metab (Lond) Springer. 2024;21:92. 10.1186/s12986-024-00859-z PMC11566664 39543676

[5] Khandelwal S, Kurpad A. A vision for nutrition research in Asia. Food Nutr Bull United States. 2019;40:133–42. 10.1177/0379572119851637 PMC7360430 31216897

[6] Shim J-S, Shim SY, Cha H-J, Kim J, Kim HC. Association between Ultra-processed food consumption and dietary intake and diet quality in Korean adults. J Acad Nutr Diet United States. 2022;122:583–94. 10.1016/j.jand.2021.07.012 34463621

[7] Pan W-H, Wu S-Y, Yeh N-H, Hung S-Y. Healthy Taiwanese eating approach (TEA) toward total wellbeing and healthy longevity. Nutrients. Switzerland; 2022. p. 14. 10.3390/nu14132774 PMC9268716 35807954

[8] Chuang S-Y, Chang H-Y, Fang H-L, Lee S-C, Hsu Y-Y, Yeh W-T, et al. The healthy Taiwanese eating approach is inversely associated with all-cause and cause-specific mortality: A prospective study on the nutrition and health survey in Taiwan, 1993–1996. PLoS One United States. 2021;16:e0251189. 10.1371/journal.pone.0251189 PMC8101962 33956833

[9] Pan W-H, Yeh N-H, Yang R-Y, Lin W-H, Wu W-C, Yeh W-T et al. Vegetable, fruit, and phytonutrient consumption patterns in Taiwan. J Food Drug Anal. China (Republic: 1949-); 2018;26:145–53. 10.1016/j.jfda.2016.12.015 PMC9332634 29389550

[10] Angeles-Agdeppa I, Sun Y, Denney L, Tanda KV, Octavio RAD, Carriquiry A, et al. Food sources, energy and nutrient intakes of adults: 2013 Philippines National nutrition survey. Nutr J Engl. 2019;18:59. 10.1186/s12937-019-0481-z PMC6785859 31601200

[11] Liu Z, Zhao L, Man Q, Wang J, Zhao W, Zhang J. Dietary micronutrients intake status among Chinese elderly people living at home: data from CNNHS 2010–2012. Nutrients Switz; 2019;11:1787. 10.3390/nu11081787 PMC6722721 31382399

[12] Mark HE, Houghton LA, Gibson RS, Monterrosa E, Kraemer K. Estimating dietary micronutrient supply and the prevalence of inadequate intakes from National food balance sheets in the South Asia regiona. Asia Pac J Clin Nutr China. 2016;25:368–76. 10.6133/apjcn.2016.25.2.11 27222421

[13] Nozue M, Ishikawa M, Takemi Y, Kusama K, Fukuda Y, Yokoyama T, et al. Prevalence of inadequate nutrient intake in Japanese Community-Dwelling older adults who live alone. J Nutr Sci Vitaminol (Tokyo) Japan. 2016;62:116–22. 10.3177/jnsv.62.116 27264096

[14] Lo Y-C, Hsu W-C, Weng S-J, Tsai Y-T, Liu S-C, Lin C-H. Event History Analysis of Factors Affecting Survival of Older Adults in Taiwan. Healthcare (Basel). Switzerland; 2022;10. 10.3390/healthcare10122439 PMC9777609 36553963

[15] Wu S-Y, Lee S-C, Yeh N-H, Wang C-F, Hung S-Y, Wu S-J, et al. Dietary characteristics of elders with frailty and with mild cognitive impairment: Cross-Sectional findings and implications from the nutrition and health survey in Taiwan 2014–2017. Switzerland: Nutrients; 2022. p. 14. 10.3390/nu14245216 PMC9782975 36558375

[16] Inui T, Hanley B, Tee ES, Nishihira J, Tontisirin K, Dael PV et al. The role of micronutrients in ageing asia: what can be implemented with the existing insights. Nutrients Switz; 2021;13:2222. 10.3390/nu13072222 PMC8308403 34209491

[17] Chen S-Y, Lin J-R, Chen T-H, Guo S-G, Kao M-D, Pan W-H. Dietary supplements usage among elderly Taiwanese during 2005–2008. Asia Pac J Clin Nutr China. 2011;20:327–36. 21669602

[18] Huang L, Yoo H-J, Abe S, Yoon J. Dietary supplement use and its related factors among Chinese international and Korean college students in South Korea. Nutr Res Pract Korea (South). 2023;17:341–55. 10.4162/nrp.2023.17.2.341 PMC10042707 37009134

[19] Huskisson E, Maggini S, Ruf M. The role of vitamins and minerals in energy metabolism and well-being. J Int Med Res Engl. 2007;35:277–89. 10.1177/147323000703500301 17593855

[20] Ji X, Grandner MA, Liu J. The relationship between micronutrient status and sleep patterns: a systematic review. Public Health Nutr Engl. 2017;20:687–701. 10.1017/S1368980016002603 PMC5675071 27702409

[21] Kaplan BJ, Crawford SG, Field CJ, Simpson JSA. Vitamins, minerals, and mood. Psychol Bull United States. 2007;133:747–60. 10.1037/0033-2909.133.5.747 17723028

[22] Long S-J, Benton D. Effects of vitamin and mineral supplementation on stress, mild psychiatric symptoms, and mood in nonclinical samples: a meta-analysis. Psychosom Med United States. 2013;75:144–53. 10.1097/PSY.0b013e31827d5fbd 23362497

[23] Abolfathi M, Pasdar Y, Kheiri M, Irandoost SF, Darabi F. The effect of consuming multivitamin/mineral supplements on elderly quality of life: based on randomized control trial. J Educ Health Promot India. 2021;10:63. 10.4103/jehp.jehp_129_20 PMC8057174 34084810

[24] Lin J-R, Lin Y-S, Kao M-D, Yang Y-H, Pan W-H. Use of supplements by Taiwanese adults aged 19–44 during 2005–2008. Asia Pac J Clin Nutr China. 2011;20:319–26. 21669601

[25] Lara-Cabrera ML, Betancort M, Muñoz-Rubilar A, Rodríguez-Novo N, Bjerkeset O, Cuevas CDL. Psychometric properties of the WHO-5 Well-Being index among nurses during the COVID-19 pandemic: A Cross-Sectional study in three countries. Int J Environ Res Public Health Switz; 2022;19:10106. 10.3390/ijerph191610106 PMC9407690 36011741

[26] Al-Naggar RA, Chen R. Prevalence of vitamin-mineral supplements use and associated factors among young Malaysians. Asian Pac J Cancer Prev Thail. 2011;12:1023–9. 21790245

[27] Utari ARD, Chairun W, Ari KS. Demographics and factor associated with food supplements use among Yogyakarta population. Int J Pharm Pharm Sci. 2017;53–6.

[28] Watcharasa P, Wongsa L, Nopparat S, Panha SK. Health literacy and dietary supplement consumption among Northeasterners of Thailand. Indian J Public Health Res Dev. 2020;11:1482–7.

[29] Versprille LJFW, Loo AJAEvd, Mackus M, Arnoldy L, Sulzer TAL, Vermeulen SA et al. Development and validation of the immune status questionnaire (ISQ). Int J Environ Res Public Health Switz. 2019;16:4743. 10.3390/ijerph16234743 PMC6926937 31783555

[30] Hays RD, Sherbourne CD, Mazel RM. The RAND 36-Item health survey 1.0. Health Econ Engl. 1993;2:217–27. 10.1002/hec.4730020305 8275167

[31] The World Health Organization-Five Well-Being Index (WHO-5). Accessed: 16 January 2025. https://www.who.int/publications/m/item/WHO-UCN-MSD-MHE-2024.01.

[32] Su Y, Cochrane BB, Reding K, Herting JR, Tinker LF, Zaslavsky O. Mediterranean diet and fatigue among Community-Dwelling postmenopausal women. J Nutr Gerontol Geriatr United States. 2022;41:22–45. 10.1080/21551197.2022.2025972 PMC9835016 35038968

[33] Cochran WG. (1977) Sampling Techniques. 3rd Edition, John Wiley & Sons, New York.

[34] Michael MJ, F BD et al. M BP, J CR, D GR, R HW,. National Institutes of Health State-of-the-Science Conference Statement: multivitamin/mineral supplements and chronic disease prevention. American Journal of Clinical Nutrition. 2007;85:257S–64S. 10.1093/ajcn/85.1.257S 17209206

[35] Michels AJ, Butler JA, Uesugi SL, Lee K, Frei BB, Bobe G, et al. Multivitamin/Multimineral supplementation prevents or reverses decline in vitamin biomarkers and cellular energy metabolism in healthy older men: A Randomized, Double-Blind, Placebo-Controlled study. Volume 15. Switzerland: Nutrients; 2023. 10.3390/nu15122691 PMC10301451 37375594

[36] Kennedy DO. B Vitamins and the Brain: Mechanisms, Dose and Efficacy–A Review. Nutrients. Switzerland; 2016;8:68. 10.3390/nu8020068 PMC4772032 26828517

[37] Dodd FL, Kennedy DO, Stevenson EJ, Veasey RC, Walker K, Reed S, et al. Acute and chronic effects of multivitamin/mineral supplementation on objective and subjective energy measures. Nutr Metab (Lond) Engl. 2020;17:16. 10.1186/s12986-020-00435-1 PMC7038616 32123534

[38] Dattola A, Silvestri M, Bennardo L, Passante M, Scali E, Patruno C, et al. Role of vitamins in skin health: a systematic review. Curr Nutr Rep United States. 2020;9:226–35. 10.1007/s13668-020-00322-4 32602055

[39] Pullar JM, Carr AC, Vissers MCM. The roles of vitamin C in skin health. Nutrients Switz; 2017;9:866. 10.3390/nu9080866 PMC5579659 28805671

[40] Draelos ZD. An oral supplement and the Nutrition-Skin connection. J Clin Aesthet Dermatol United States. 2019;12:13–6. PMC6715334 31531157

[41] Esquivel MK, Ghosn B. Current evidence on common dietary supplements for sleep quality. Am J Lifestyle Med United States. 2024;18:323–7. 10.1177/15598276241227915 PMC11082867 38737872

[42] Haskell CF, Robertson B, Jones E, Forster J, Jones R, Wilde A, et al. Effects of a multi-vitamin/mineral supplement on cognitive function and fatigue during extended multi-tasking. Hum Psychopharmacol Engl. 2010;25:448–61. 10.1002/hup.1144 20737518

[43] White DJ, Cox KHM, Peters R, Pipingas A, Scholey AB. Effects of Four-Week supplementation with a Multi-Vitamin/Mineral Preparation on mood and blood biomarkers in young adults: A Randomised, Double-Blind, Placebo-Controlled trial. Nutrients Switz. 2015;7:9005–17. 10.3390/nu7115451 PMC4663579 26529011

[44] Yeung L-K, Alschuler DM, Wall M, Luttmann-Gibson H, Copeland T, Hale C, et al. Multivitamin supplementation improves memory in older adults: A randomized clinical trial. Am J Clin Nutr United States. 2023;118:273–82. 10.1016/j.ajcnut.2023.05.011 PMC10375458 37244291

[45] Baker LD, Manson JE, Rapp SR, Sesso HD, Gaussoin SA, Shumaker SA, et al. Effects of cocoa extract and a multivitamin on cognitive function: A randomized clinical trial. Alzheimers Dement United States. 2023;19:1308–19. 10.1002/alz.12767 PMC10011015 36102337

[46] Vyas CM, Manson JE, Sesso HD, Cook NR, Rist PM, Weinberg A, et al. Effect of multivitamin-mineral supplementation versus placebo on cognitive function: results from the clinic subcohort of the cocoa supplement and multivitamin outcomes study (COSMOS) randomized clinical trial and meta-analysis of 3 cognitive studies within COSMOS. Am J Clin Nutr United States. 2024;119:692–701. 10.1016/j.ajcnut.2023.12.011 PMC11103094 38244989

[47] Carroll D, Ring C, Suter M, Willemsen G. The effects of an oral multivitamin combination with calcium, magnesium, and zinc on psychological well-being in healthy young male volunteers: a double-blind placebo-controlled trial. Psychopharmacol Ger. 2000;150:220–5. 10.1007/s002130000406 10907676

[48] Barringer TA, Kirk JK, Santaniello AC, Foley KL, Michielutte R. Effect of a multivitamin and mineral supplement on infection and quality of life. A randomized, double-blind, placebo-controlled trial. Ann Intern Med United States. 2003;138:365–71. 10.7326/0003-4819-138-5-200303040-00005 12614088

[49] Munteanu C, Schwartz B. The relationship between nutrition and the immune system. Front Nutr Switz. 2022;9:1082500. 10.3389/fnut.2022.1082500 PMC9772031 36570149

[50] Fantacone ML, Lowry MB, Uesugi SL, Michels AJ, Choi J, Leonard SW, et al. The effect of a multivitamin and mineral supplement on immune function in healthy older adults: A Double-Blind, Randomized, controlled trial. Nutrients. Switzerland; 2020. p. 12. 10.3390/nu12082447 PMC7468989 32823974

[51] European Commission Commission Regulation (EU). 432/2012 of 16 May 2012. Establishing a list of permitted health claims made on foods, other than those referring to the reduction of disease risk and to children’s development and health. O J Eur Un. 2012. http://data.europa.eu/eli/reg/2012/432/2025-08-20

[52] Blome C, Augustin M. Measuring change in quality of life: bias in prospective and retrospective evaluation. Value Health United States. 2015;18:110–5. 10.1016/j.jval.2014.10.007 25595241

[53] Jager KJ, Tripepi G, Chesnaye NC, Dekker FW, Zoccali C, Stel VS. Where to look for the most frequent biases? Nephrology (Carlton). Australia. 2020;25:435–41. 10.1111/nep.13706 PMC7318122 32133725

