# Supplementary material for: The role of multivitamin and mineral supplements in supporting health and well-being: a retrospective cross-sectional study in Taiwan
Source: J Health Popul Nutr. 2025 Dec 23;44:423. doi: 10.1186/s41043-025-01164-y (PMC12729314; doi:10.1186/s41043-025-01164-y)
Supplement: Supplementary file 1 — Supplementary Material 1 [file 41043_2025_1164_MOESM1_ESM.docx]

**Supplementary Material**

**for**

**The Role of Multivitamin and Mineral Supplements in Supporting Health and Well-Being: A Retrospective Cross-Sectional Study in Taiwan**

**Vandana Garg^1^*, Joseph Lin^2^, Aida Gadzhieva-Moore^3^, Shi Mun Yee^4^, Abhijeet Dhiman^5^, Richa Kumari^5^, Sheryl S.L. Tan^1^**

^1^ Haleon, 23 Rochester Park, Singapore, 139234

^2^ Haleon, UK Services Limited Taiwan Branch, Taipei, Taiwan, 100507

^3^ IQVIA Solutions Asia Pte Ltd, 79 Anson Road, Singapore, 079906

^4^ IQVIA Solutions Malaysia Sdn Bhd, No. 1, Jalan SS21/58, Damansara Uptown, 47400 Petaling Jaya, Malaysia

^5^ Knowledge Centre, WNS Global Services, Gurugram, India, 122002

* Correspondance: Vandana Garg: vandana.x.garg@haleon.com

**Table of contents**

[**Supplementary Table S1: Demographics and other characteristics** 3](#_Toc190422116)

[**Supplementary Table S2**: **Youthful vitality benefit assessment** 4](#_Toc190422117)

[**Supplementary Table S3**: **Energy and performance-related benefits** 5](#_Toc190422118)

[**Supplementary Table S4: Detailed answers of the SF-36 vitality scales** 6](#_Toc190422119)

[**Supplementary Table S5**: **Vitality scores** 7](#_Toc190422120)

[**Supplementary Table S6**: **Metabolism-related benefits** 8](#_Toc190422121)

[**Supplementary Table S7**: **Skin health and complexion-related benefits** 9](#_Toc190422122)

[**Supplementary Table S8**: **Immunity status and WHO-5 well-being score** 10](#_Toc190422123)

[**Supplementary Table S9**: **QoL-related benefits** 11](#_Toc190422124)

[**Supplementary Table S10**: **Attitude and perception.** 12](#_Toc190422125)

**Supplementary Table S1: Demographics and other characteristics**

Data shown in total level (n = 400) for existing MVM supplements users.

| **Characteristics** | **Percentage respondents (%), n = 400** |
| --- | --- |
| Gender | |
| Male | 50.25% |
| Female | 49.75% |
| Age (Years) | |
| <50 (35-49) | 64.25% |
| ≥50 (50-60) | 35.75% |
| Frequency of use | |
| Daily user | 59.25% |
| Occasion user (3–5 days per week) | 40.75% |
| Monthly household income (NT$) | |
| <30,000 | 8.00% |
| 30,001-70,000 | 29.00% |
| 70,001-90,000 | 13.75% |
| 90,001-110,000 | 18.00% |
| 110,001-130,000 | 11.25% |
| 130,001-150,000 | 7.50% |
| ≥150,001 | 12.50% |

**Supplementary Table S2**: **Youthful vitality benefit assessment**

Data based on 400 respondents' perceived youthful vitality benefits from taking MVM supplements. Respondents were asked to select which of the six listed youthful vitality benefits they perceived from taking MVM supplements or 'None of the above' if they experienced none. Since multiple selections were allowed, 93% of respondents reported at least one benefit, while 7% chose 'None of the above'. The percentages for each benefit represent the proportion of respondents who reported experiencing that specific effect.

| Benefits | Percentage respondents (%),  n = 400 |
| --- | --- |
| With MVM supplements, I can maintain a good spirit | 53.25% |
| With MVM supplements, I can maintain good overall complexion | 35.50% |
| With MVM supplements, I can have good energy | 55.75% |
| With MVM supplements, I can maintain my mental alertness | 22.25% |
| MVM supplements help me fall asleep easier | 17.50% |
| With MVM supplements, I can have a good metabolism | 33.50% |
| None of the above | 7.00% |
| NET: Respondents who perceived at least 1 out of 6 youthful vitality benefits (e.g., energy, spirit, complexion, metabolism, sleep and mental alertness) | 93.00% |

**Supplementary Table S3**: **Energy and performance-related benefits**

Data based on 400 respondents’ assessment on their experience pertaining to the benefits of MVM supplements on energy levels and performance, and the aggregated percentage score of those who chose ‘Agree’ and ‘Strongly agree’. A 5-point scale was employed. Subgroup comparison between male vs female, <50-year-old vs ≥50-year-old users and daily vs occasional users.

| Energy and performance-related benefits | Total (n = 400) | Male (n = 201) | Female (n = 199) | <50 year-old (n = 257) | ≥50 year-old (n = 143) | Daily user (n = 237) | Occasional user (n = 163) |
| --- | --- | --- | --- | --- | --- | --- | --- |
| MVM supplements give me good energy all day long. | 49.00% | 54.23%* | 43.72%* | 47.08% | 52.45% | 50.21% | 47.24% |
| MVM supplements gives me energy to perform better at my day-to-day life. | 59.00% | 63.18% | 54.77% | 56.81% | 62.94% | 56.12% | 63.19% |
| With MVM supplements, I feel more energetic to engage in my personal hobbies. | 47.75% | 50.75% | 44.72% | 45.53% | 51.75% | 48.10% | 47.24% |
| With MVM supplements, I find I have more energy for sports/ training. | 50.50% | 55.22% | 45.73% | 48.25% | 54.55% | 54.43% | 44.79% |

*p = 0.034

**Supplementary Table S4: Detailed answers of the SF-36 vitality scales**

Data based on 400 respondents’ perceived frequency of experience for each statement pertaining to their energy levels in the past 4 weeks, and the aggregated percentage score of those who chose ‘All of the time’, ‘Most of the time’ and ‘A good bit of the time’.

| **SF-36 vitality scales items** | **Top 3 Box** | **All of the time** | **Most of the time** | **A good bit of the time** | **Some of the time** | **A little of the time** | **None of the time** |
| --- | --- | --- | --- | --- | --- | --- | --- |
| Did you feel full of pep? | 42.50% | 3.50% | 16.25% | 22.75% | 33.75% | 23.00% | 0.75% |
| Did you have a lot of energy? | 39.00% | 3.00% | 16.50% | 19.50% | 34.25% | 25.25% | 1.50% |
| Did you feel worn out? | 28.50% | 2.25% | 7.50% | 18.75% | 44.50% | 25.50% | 1.50% |
| Did you feel tired? | 35.00% | 4.25% | 7.75% | 23.00% | 47.00% | 17.75% | 0.25% |

**Supplementary Table S5**: **Vitality scores**

Data shown in total level (n = 400), and with subgroup comparison between male vs female, <50-year-old vs ≥50-year-old users and daily vs occasional users.

| **Vitality score (score range 0 to 100)** | **Total (n = 400)** | **Male (n = 201)** | **Female (n = 199)** | **<50 year-old (n = 257)** | **≥50 year-old (n = 143)** | **Daily user (n = 237)** | **Occasional user (n = 163)** |
| --- | --- | --- | --- | --- | --- | --- | --- |
| **Mean Vitality Score** | 51.48 | 52.96 | 49.97 | 49.92* | 54.27* | 52.87^$^ | 49.45^$^ |
| **Standard Deviation** | 17.84 | 18.70 | 16.84 | 17.19 | 18.69 | 19.44 | 15.05 |

*p = 0.023 and ^$^p = 0.048

**Supplementary Table S6**: **Metabolism-related benefits**

Data based on 400 respondents’ assessment on their experience pertaining to the benefits of MVM supplements on metabolism, and the aggregated percentage score of those who chose ‘Agree’ and ‘Strongly agree’. A 5-point scale was employed. Subgroup comparison between male vs female, <50-year-old vs ≥50-year-old users and daily vs occasional users.

| **Metabolism-related benefits** | **Total (n = 400)** | **Male (n = 201)** | **Female (n = 199)** | **<50 year-old (n = 257)** | **≥50 year-old (n = 143)** | **Daily user (n = 237)** | **Occasional user (n = 163)** |
| --- | --- | --- | --- | --- | --- | --- | --- |
| With MVM supplements, my metabolism has improved. | 59.75% | 65.17%* | 54.27%* | 56.42% | 65.73% | 61.18% | 57.67% |

*p = 0.025

**Supplementary Table S7**: **Skin health and complexion-related benefits**

Data based on 400 respondents’ assessment on their experience pertaining to the benefits of MVM supplements on skin health and complexion, and the aggregated percentage score of those who chose ‘Agree’ and ‘Strongly agree’. A 5-point scale was employed. Subgroup comparison between male vs female, <50-year-old vs ≥50-year-old users and daily vs occasional users.

| **Skin health and complexion statements** | **Total (n = 400)** | **Male (n = 201)** | **Female (n = 199)** | **<50 year-old (n = 257)** | **≥50 year-old (n = 143)** | **Daily user (n = 237)** | **Occasional user (n = 163)** |
| --- | --- | --- | --- | --- | --- | --- | --- |
| MVM supplements improves my overall complexion. | 53.25% | 55.72% | 50.75% | 49.42%* | 60.14%* | 51.05% | 56.44% |
| My skin looks better while taking MVM supplements. | 38.00% | 37.81% | 38.19% | 33.85%^$^ | 45.45%^$^ | 38.40% | 37.42% |
| My skin looks healthier while taking MVM supplements. | 36.25% | 35.32% | 37.19% | 34.24% | 39.86% | 35.44% | 37.42% |
| MVM supplements improves my overall skin health. | 36.50% | 34.83% | 38.19% | 33.07% | 42.66% | 35.44% | 38.04% |

*p = 0.037 and ^$^p = 0.023

**Supplementary Table S8**: **Immunity status and WHO-5 well-being score**

Data presented for total (n = 400) and subgroup (male vs female, <50-year-old vs ≥50-year-old users and daily vs occasional users).

|  | **Total (n = 400)** | **Male (n = 201)** | **Female (n = 199)** | **<50 year-old (n = 257)** | **≥50 year-old (n = 143)** | **Daily user (n = 237)** | **Occasional user (n = 163)** |
| --- | --- | --- | --- | --- | --- | --- | --- |
| **Immunity status (ISQ derived), score range 0 to 10** | | | | | | | |
| Normal immune functioning  (ISQ ≥6) | 66.25% | 69.15% | 63.32% | 66.15% | 66.43% | 69.20% | 61.96% |
| Reduced immune functioning (ISQ <6) | 33.75% | 30.85% | 36.68% | 33.85% | 33.57% | 30.80% | 38.04% |
| Mean ISQ score | 6.44 | 6.78* | 6.09* | 6.24^$^ | 6.80^$^ | 6.49 | 6.36 |
| Standard Deviation | 2.22 | 2.31 | 2.06 | 2.20 | 2.21 | 2.29 | 2.11 |
| **WHO-5 well-being score, score range 0 to 25** | | | | | | | |
| Normal mental well-being (WHO-5 score ≥13) | 58.00% | 58.71% | 57.29% | 55.64% | 62.24% | 63.29% | 50.31% |
| Poor mental well-being (WHO-5 score <13) | 42.00% | 41.29% | 42.71% | 44.36% | 37.76% | 36.71% | 49.69% |
| Mean WHO-5 Score | 13.23 | 13.51 | 12.95 | 12.84** | 13.94** | 13.89^$$^ | 12.26^$$^ |
| Standard Deviation | 5.20 | 5.22 | 5.17 | 5.16 | 5.21 | 5.45 | 4.65 |

*p = 0.002, **^$^** p = 0.016; ** p = 0.043, p^$$^ = 0.001

**Supplementary Table S9**: **QoL-related benefits**

Data based on 400 respondents’ assessment on their experience pertaining to the benefits of MVM supplements on quality of life (QoL), and the aggregated percentage score of those who chose ‘Agree’ and ‘Strongly agree’. A 5-point scale was employed. Subgroup comparison between male vs female, <50-year-old vs ≥50-year-old users and daily vs occasional users.

| **QoL-related statements** | **Total (n = 400)** | **Male (n = 201)** | **Female (n = 199)** | **<50 year-old (n = 257)** | **≥50 year-old (n = 143)** | **Daily user (n = 237)** | **Occasional user (n = 163)** |
| --- | --- | --- | --- | --- | --- | --- | --- |
| My quality of life improved with MVM supplements. | 49.50% | 56.22%* | 42.71%* | 47.86% | 52.45% | 48.10% | 51.53% |
| With MVM supplements, I am taking good care of my body and mind. | 60.50% | 65.17% | 55.78% | 57.59% | 65.73% | 57.81% | 64.42% |
| MVM supplements supports me to keep a healthy and active lifestyle and enjoy activities I love. | 53.75% | 59.20% | 48.24% | 52.53% | 55.94% | 54.43% | 52.76% |
| With just 1 pill a day, MVM supplements gives me confidence to maintain my overall health. | 63.25% | 67.66% | 58.79% | 61.87% | 65.73% | 61.18% | 66.26% |

*p = 0.006

**Supplementary Table S10**: **Attitude and perception.**

Data shown in total level (n = 400) for existing users’ attitude and perception towards MVM supplements.

| **Trust** | |
| --- | --- |
| *Top 2 Box* | 72.50% |
| I trust it | 66.00% |
| I completely trust it | 6.50% |
| I am not sure whether I trust it or not | 25.75% |
| I do not trust it | 1.50% |
| I do not trust it at all | 0.25% |
| **Overall Satisfaction** | |
| *Top 2 Box* | 74.50% |
| Satisfied | 68.25% |
| Completely satisfied | 6.25% |
| Neither satisfied nor dissatisfied | 24.50% |
| Dissatisfied | 1.00% |
| Absolutely dissatisfied | - |
| **Continued Purchase** | |
| *Top 2 Box* | 79.00% |
| I would buy it again | 66.25% |
| I would definitely buy it again | 12.75% |
| I am not sure whether I would buy it again or not | 19.50% |
| I would not buy it again | 1.25% |
| I would definitely not buy it again | 0.25% |
| **Likelihood to Recommend (Others)** | |
| *Top 2 Box* | 63.75% |
| I would recommend it to others | 56.25% |
| I would definitely recommend it to others | 7.50% |
| I am not sure whether I would recommend it or not to others | 32.25% |
| I would not recommend it to others | 4.00% |
| I would definitely not recommend it to others | - |
| **Likelihood to Recommend (Friends & Family)** | |
| *Top 2 Box* | 68.75% |
| I would recommend it | 61.25% |
| I would definitely recommend it | 7.50% |
| I am not sure whether I would recommend it or not | 28.50% |
| I would not recommend it | 2.75% |
| I would definitely not recommend it | - |
